# The role of postoperative radiotherapy in patients with uterine sarcomas: A PSM-IPTW analysis based on SEER database

**DOI:** 10.3389/fsurg.2022.985654

**Published:** 2022-08-09

**Authors:** Zhimin Hao, Sufen Yang

**Affiliations:** ^1^ Department of Gynecology, The Affiliated Hospital of Medical School of Ningbo University, Ningbo, China; ^2^ School of Medicine, Ningbo University, Ningbo, China

**Keywords:** uterine sarcomas, PSM, IPTW, leiomyosarcoma, radiotherapy, chemotherapy methods study population

## Abstract

**Objective:**

The optimal adjuvant therapy for uterine sarcomas remains poorly determined due to its rarity and histological diversity. The purpose of the study is to explore and characterize the association between utilization of radiotherapy and survival outcome in patients with surgically resected uterine sarcomas.

**Methods:**

We collected data regarding uterine sarcomas which were confirmed after total hysterectomy between 2010 and 2018 period from the latest version of the Surveillance, Epidemiology, and End Results (SEER) database. Initially, 1-, 3- and 5-year survival rate were calculated to predict potential risk factors and possible role of adjuvant chemotherapy and radiotherapy. Propensity score matching (PSM) and inverse probability treatment weighting (IPTW) technique were employed to balance confounding factors in the utilization of additional therapy. Multivariate and exploratory subgroup analyses were respectively conducted to evaluate the impact of adjuvant therapy on overall survival (OS) and cause-specific survival (CSS).

**Results:**

A total of 2897 patients were enrolled in the analysis. Survival benefit at 1-, 3-and 5-year after initial treatment was observed in the group of radiotherapy given, however, poorer prognosis in the group of chemotherapy administration. Accordingly, chemotherapy was enrolled as a confounding factor when stratifying and matching patients by receipt of radiotherapy. Prior to and after PSM-IPTW adjustment, radiotherapy both demonstrated beneficial effect on OS and CSS based on multivariate analysis. Further subgroup analysis indicated radiotherapy improved OS and CSS among a subset of patients in stage II-IV, particularly with uterine leiomyosarcoma, tumor grade IV, bigger tumor size than 100 mm and even with chemotherapy administration.

**Conclusions:**

Adjuvant radiotherapy in uterine sarcomas after hysterectomy might be underutilized, and proper use of adjuvant radiotherapy combined with chemotherapy after surgery in advanced-stage and high-risk patients might improve survival.

## Introduction

Uterine sarcomas (US) are a heterogeneous and rare group of neoplasms, accounting for approximately 1% of female genital tract malignancies and 3%–7% of all uterine neoplasms (1). The most represented histological subtype of uterine sarcomas is leiomyosarcoma (LMS), followed subsequently by endometrial stromal sarcoma (ESS), adenosarcoma and undifferentiated uterine sarcoma (USS) (2). Low-grade ESS and high-grade ESS represents approximately 86% and 14%, respectively, of all endometrial stromal sarcomas as described in a relatively large study regarding uterine mesenchymal tumors (3). Uterine carcinosarcoma has been excluded from US division, instead, as a subtype of high-grade endometrial carcinoma (4).

Total hysterectomy remains the standard surgery mode for newly diagnosed early-stage uterine sarcomas, often in combination with bilateral salpingo- oophorectomy, and generous tumor debulking if present outside the uterus (5). Although lymphadenectomy may confer more accurate staging, it was not related to a clear survival benefit in patients with uterine sarcomas (6). It's worth noting that the uncontained power morcellation during laparoscopic hysterectomy or myomectomy has become as a popular surgical modality for tissue extraction within past decades. In recent years, where several cases of abdominopelvic dispersion developing from electrical morcellation of unexpected uterine sarcomas were reported in term of severely negative repercussions (7), clinicians paid more attention to its impact on US patients' outcomes and survival. It was commonly recognized that women whose malignant tumor tissue was unintended morcellated at time of hysterectomy or myomectomy posed a higher risk of distant recurrence as compared to local recurrence presumed to be attributed to intra-abdominal seeding or lymphvascular spread of small volume specimen at time of *en bloc* hysterectomy (8). Even in the setting of localized disease, the 5-year survival rate for US is 50%–75%. Prognosis of those in advanced stage disease is poorer, with the 5-year survival probability of approximately 30%–45% (9). The high rate of recurrence and poor prognosis, particularly for LMS (10) and high grade ESS (3, 11), provides the rationale for evaluation of adjuvant therapy to improve prognosis. Given its rarity and histological diversity, it is difficult to reach consensus concerning the best route of adjuvant radiotherapy or chemotherapy through prospective clinical trials (12). Hence, large-database retrospective analysis utilizing the tools currently available, such as SEER, can still help tailor clinical practice and inform investigation of future treatments.

Moreover, individual differences in patients' response to adjuvant therapy are of key interest in clinical practice. In light of this, some important clinical parameters, such as age, race, tumor grade and size, are usually considered when predicting survival outcomes. For instance, black women experienced a higher risk of uterine sarcoma than those white females, as well as women aged over 50 years posed a higher risk of sarcoma (13). Accordingly, balancing the potential confounding factors is necessary to improve the accuracy of survival prediction among women with uterine sarcomas.

The purpose of the current study is to comprehensively explore and characterize the association between utilization of radiotherapy and survival outcome in patients with surgically resected uterine sarcomas. Besides, we evaluate other variables for their prognostic significance in uterine sarcomas.

## Methods

### Study population

We conducted a retrospective analysis for patients with uterine sarcomas between 2010 and 2018. The Surveillance, Epidemiology and End Results (SEER) database (SEER*Stat 8.3.9.2), which contains data of cancer patients from 18 regional registries (https://seer.cancer.gov/seerstat/), was employed for the analysis. Uterine sarcomas were confirmed by histology of hysterectomy specimen and based on the WHO International Classification of Diseases for Oncology, third edition (ICD-O-3) morphology codes as follows: Leiomyosarcoma: 8890-leiomyosarcoma, NOS, 8891-epithelioid leiomyosarcoma, 8896-myxoid leiomyosarcoma; Endometrial stromal sarcoma: 8930-endometrial stromal sarcoma, 8931-endometrial stromal sarcoma, low-grade; 8935-Stromal sarcoma, NOS; Undifferentiated uterine sarcoma: 8805; Adenosarcoma: 8933. Based on site-specific surgery codes, women who underwent total hysterectomy with or without bilateral salpingoopherectomy were selected, including those with modified or radical hysterectomy. We excluded those cases with the surgery code “local tumor excision or destruction; subtotal hysterectomy; surgery NOS” given the fact that we could not identify the scope of the surgical procedure performed. Since all data included in the SEER database is publicly available online, this study does not require Institutional Review Board approval, or informed consent by the study subjects. While, we obtained permission to access the SEER program data from the US National Cancer Institute (reference number: 22756-Nov2020).

**Figure 1:**
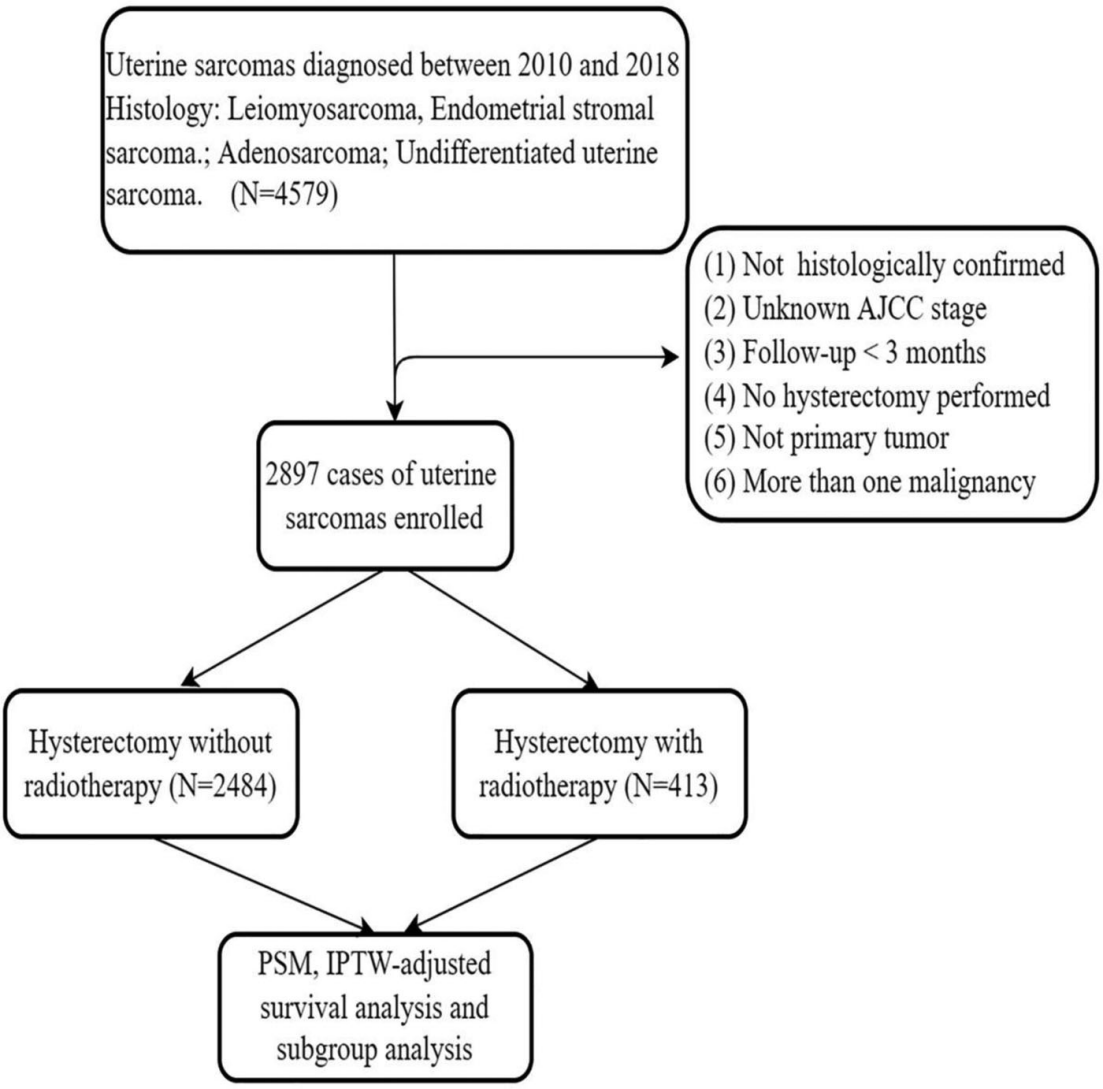
Eligibility, inclusion, and exclusion criteria of the study population.

Those cases with more than one malignancy or secondary tumor, missing information on age, stage, unknown survival period or not hysterectomy performed were excluded from the analysis. A landmark survival time of 3 months was applied in order to account for immortal time bias. These procedures were demonstrated as detailed in the diagram Figure 1.

### Variable record and cohort definition

Demographic information of the patients encompassed age (<50, 50–60, >60), year of diagnosis (2010–2012, 2013–2015, 2016–2018), marital status (married, single, divorced, widowed), race (white, black, others), urban or rural area patients lived and median household income. Tumor characteristics included stage (I, II, III and IV), grade (grade I, well differentiated; grade II, moderately differentiated; grade III, poorly differentiated; grade IV, undifferentiated; unknown grade), tumor size (<50 mm, 50–100 mm, >100 mm, unknown), peritoneal cytology (negative, positive or unknown). Treatment data involved lymphadenectomy (yes, none or unknown), months from diagnosis to treatment, radiotherapy (external beam, brachytherapy, combined of both or none) and chemotherapy (yes, none or unknown).

The primary endpoints were 1-, 3-and 5-year overall survival (OS), as well as the corresponding cause specific survival (CSS). The definition of OS was the time from confirmed diagnosis to death for any cause or to date of last follow-up, and CSS was defined as the interval from final diagnosis to death due to uterine sarcomas. Data from patients alive at the last visit were censored.

### Statistical analysis

Categorical variables are shown as frequency and continuous variables are described as median (interquartile range [IQR]). Baseline patient characteristics were compared both pre- and post-matching with Chi-square test analysis, where the statistical significance in proportions' differences with *P* value <0.05 was identified unbalanced. To investigate impact of radiotherapy on survival in US patients, multiple imputations by chained equations were conducted to reduce potential bias resulted from missing data. First, we used a propensity score adjustment by inverse probability of treatment-weighting (IPTW) to maximally reduce the differences between radiotherapy and no radiotherapy administration, as previously described (14, 15). Specifically, the propensity score was calculated using a logistic regression model based on the abovementioned characteristics. Stratified by radiotherapy administrated or not, propensity score matching (PSM) method (16) was employed through the nearest neighbor-matching with caliper value 0.4 for 1:4 matching. Afterwards, IPTW was calculated as 1/PS in the group of radiotherapy given, whereas 1/(1-PS) in the cohort without radiotherapy administered (17). Stabilization of the IPTW was performed by multiplying the standard IPTW by the probability of undergoing treatment that each patient received (18). Prior to and after IPTW-adjustment, univariate analysis (UVA) of patient characteristics effect on CSS and OS was conducted using the Kaplan-Meier (KM) method, with the log-rank method for evaluation for significance. Multivariable analysis (MVA) was performed through Cox proportional hazards regression model. Covariates enrolled in the MVA model were selected if they were significant in the UVA model. Next, we conducted exploratory subgroup analyses and evaluated heterogeneity as the subgroups are presumed to have been subjected to similar conditions (19). Quantification of heterogeneity was evaluated with the I^2^ statistic and the Cochran Q test (20). Random-effects models were used when study heterogeneity was high (I^2^ > 50%) and fixed-effects models were employed whereas heterogeneity was low (I^2^ ≤ 50%) (21). Finally, IPTW-adjusted Kaplan-Meier plots illustrated CSS rates based on radiotherapy administration or not in some selected subgroups. Statistical analyses were executed with SPSS (version 22.0, SPSS, Chicago, IL, USA), R software (version 3.6.3; http://www.r-project.org/) and STATA-MP (version 17.0, College Station, TX, USA), with two-sided *P* < 0.05 considered statistically significant.

## Results

### Descriptive characteristics of the study population and survival outcome among all subgroups

According to the set criteria, a total of 2897 patients, who were diagnosed as uterine sarcomas as the primary malignancy and underwent total hysterectomy, were extracted during 2010 and 2018 period. The cohort comprised 1582 leiomyosarcomas, 862 endometrial stromal sarcomas, 404 adenosarcomas and 49 undifferentiated uterine sarcomas. 63.55% (1841/2897) of cases were present in stage I, of note, nearly 87% of uterine adenosarcoma presented with stage I disease. The median age at initial diagnosis in the whole cohort was 54 year old [interquartile range (IQR): 47-62 years old]. The median follow-up period was 33 months [interquartile range (IQR): 15-63 months]. The 1-, 3-year and 5-year CSS rates were 56.89%, 39.63% and 24.54% for the whole cohort, respectively. Meanwhile, the corresponding OS rates were 55.02%, and 38.69% and 24.23%, respectively. However, for patients diagnosed between 2016 and 2018, 5-year CSS and rates were not calculated due to the short follow period. Also, some information about surgeon who performed the surgery was not specified, as well as mode of surgery, open or minimal invasive. For patients with LMS and UUS, shorter survival period was observed compared to those with EES and adenosarcoma. Positive peritoneal cytology posed a significant poorer survival in every specific study period compared to those negative cases. Patients with lymphadenectomy showed similar survival outcomes to those without the procedure. In stage IVB patients, lung was the commonest metastatic site, while all of those with brain metastasis were dead within 1 year after initial diagnosis. Although adjuvant chemotherapy was administered in 40.25% (1166/2897) of cases, no improved survival was shown, conversely, detrimental effect on CSS and OS. Whereas radiotherapy was just administered in 14.26% (413/2897) of patients, beneficial effect was observed on CSS and OS, particularly the combination of external beam and brachytherapy. The demographic and clinical characteristics of these US patients and survival outcome in those subgroups were summarized in Table 1.

**Table 1:** The 1-, 3- and 5-year cause-specific survival and overall survival in terms of uterine sarcoma patients.

		Cause-specific survival (%)			Overall survival(%)		
Characteristics	Num	1-year	3-year	5-year	1-year	3-year	5-year
Total	2897	56.89	39.63	24.54	55.02	38.69	24.23
Age (years)							
<50	1003	69.19	49.05	31.31	68.10	48.55	31.11
50–60	1026	65.52	45.81	28.57	63.79	45.20	28.33
>60	868	48.62	32.72	19.01	45.28	30.76	18.43
Race							
Black	501	49.70	32.93	21.36	47.70	32.33	21.16
Others	327	62.69	42.51	25.38	61.16	42.51	25.38
White	2069	57.71	42.73	25.18	55.82	39.63	24.79
Marital status							
Married	1515	59.41	40.79	25.48	57.69	40.01	25.35
Divorced/separated	320	60.63	42.81	25.63	57.81	41.25	25.00
Single/unmarried	712	51.97	36.24	22.47	50.70	35.53	22.19
Unknown	151	56.95	42.38	28.48	55.63	42.38	28.48
Widowed	199	49.25	35.68	20.10	45.23	32.66	18.59
Median income							
<$50,000	339	56.64	39.82	26.84	53.39	38.05	25.66
$50,000–$65,000	998	54.61	43.29	29.16	52.71	42.28	28.76
>$65,000	1560	58.40	37.24	21.09	56.86	36.54	21.03
Year of diagnosis							
2010–2012	915	50.16	48.42	46.33	47.43	46.67	45.57
2013–2015	980	55.82	52.04	29.29	53.37	51.02	29.08
2016–2018	1002	64.07	19.36	N/A	63.57	19.36	N/A
Tumor grade							
I	544	88.05	62.87	39.55	86.58	61.76	38.97
II	223	79.37	65.47	43.95	78.03	65.02	43.50
III	389	40.87	22.62	13.37	37.79	21.33	13.11
IV	696	37.36	28.30	17.39	35.49	27.87	17.39
Unknown	1045	54.83	35.89	21.53	53.11	34.74	21.15
Histology							
Adenosarcoma	404	73.02	51.24	30.94	70.30	49.51	30.45
ESS	862	70.42	51.28	32.48	68.91	50.46	32.13
LMS	1582	45.76	30.66	18.77	44.06	29.9	18.52
UUS	49	44.90	28.57	18.37	38.78	24.49	18.37
AJCC stage							
I	1841	70.45	49.43	30.58	68.39	48.18	30.15
II	274	52.55	40.51	26.28	51.09	39.78	25.91
III	243	36.21	20.99	14.81	32.92	20.99	14.81
IV	539	23.01	14.10	7.42	21.33	13.73	7.42
Distant metastasis							
Lung	310	19.35	10.97	5.48	17.74	10.65	5.48
Liver	56	21.43	14.29	3.57	17.86	14.29	3.57
Bone	49	14.29	8.16	2.04	14.29	8.16	2.04
Brain	7	0	0	0	0	0	0
Lymphadenectomy							
Yes	1000	58.10	42.70	28.80	56.20	41.90	28.60
None/unknown	1897	56.25	38.01	22.30	54.40	37.01	21.93
Peritoneal cytology							
Negative	1095	59.54	42.37	27.12	57.17	41.19	26.76
Positive	114	27.19	21.05	13.16	23.68	18.42	13.16
Unknown	1688	57.17	39.10	23.64	55.75	38.45	23.34
Tumor size(mm)							
<50	475	78.53	56.00	34.95	76.00	54.74	34.32
50–100	1018	61.59	42.34	25.05	59.73	41.26	24.75
>100	1054	39.56	26.28	15.37	37.76	25.62	15.18
Unknown	350	66.00	49.71	36.57	64.86	48.86	36.29
Chemotherapy							
Yes	1166	35.93	23.41	12.86	33.88	22.56	12.78
None/unknown	1731	71.00	50.51	32.41	69.27	49.57	31.95
Radiotherapy							
None/unknown	2484	57.76	39.39	24.00	55.95	38.50	23.71
Beam	301	47.18	37.54	24.58	45.52	36.54	24.58
Brachytherapy	45	60.00	44.44	28.89	55.56	42.22	26.67
Combination	67	66.67	55.56	42.86	63.49	53.97	41.27

EES, endometrial stromal sarcoma; LMS, leiomyosarcoma; UUS, undifferentiated uterine sarcoma.

### Exploration of adjuvant radiotherapy utilization among subgroups

To further investigate the association of radiotherapy among various uterine sarcomas and clinicopathologic parameters, we stratified the cohort by receipt of adjuvant radiotherapy or not. Before PSM and IPTW-adjustment, most baseline characteristics were significantly unbalanced. Patients who received additional radiotherapy tended to be older than 60 years of age, diagnosed between 2010 and 2012, with tumor grade III-IV and tumor size bigger than 50 mm, in groups of AJCC stage II-IV and chemotherapy administration. After PSM and IPTW-adjustment by RT, all baseline characteristics were well balanced with *P* value >0.05. The results were demonstrated in Table 2.

**Table 2:** Baseline characteristics in uterine sarcomas before and after IPTW-adjustment according to RT.

Characteristics	Unadjusted (*n* = 2897)			IPTW-adjusted (*n* = 2886)		
	Surgery-RT(*n*, %)	Surgery + RT(*n*, %)	*P-*value	Surgery-RT	Surgry + RT	*P-*value
Age (years)			0.004*			0.417
<50	886(35.67)	117(28.33)		34.52%	31.29%	
50–60	878(35.35)	148(35.84)		35.47%	36.58%	
>60	720(28.99)	148(35.84)		30.01%	32.13%	
Year of diagnosis			0.000*			0.543
2010–2012	748(30.11)	167(40.44)		31.66%	32.55%	
2013–2015	841(33.86)	139(33.66)		33.82%	35.66%	
2016–2018	895(36.03)	107(25.91)		34.52%	31.80%	
Marital status			0.080			0.945
Married	1297(52.21)	218(52.78)		52.33%	52.68%	
Single/unmarried	622(25.04)	90(21.79)		24.55%	24.30%	
Divorced/separated	270(10.87)	50(12.11)		11.01%	11.54%	
Unknown	135(5.43)	16(3.87)		5.20%	4.21%	
Widowed	160(6.44)	39(9.44)		6.91%	7.26%	
Race			0.519			0.716
Black	428(17.23)	73(17.68)		17.30%	17.07%	
Others	274(11.03)	53(12.83)		11.35%	12.76%	
White	1782(71.74)	287(69.49)		71.35%	70.17%	
Tumor differentiation			0.000*			0.915
I	508(20.45)	36(8.72)		18.74%	16.93%	
II	197(7.93)	26(6.30)		7.70%	7.79%	
III	323(13.00)	66(15.98)		13.39%	12.98%	
IV	538(21.66)	158(38.26)		24.11%	25.42%	
Unknown	918(36.96)	127(30.75)		36.06%	36.88%	
Histology			0.032			0.648
Adenosarcoma	344(13.85)	60(14.53)		13.81%	12.14%	
ESS	746(30.03)	116(28.09)		29.79%	27.98%	
LMS	1359(54.71)	223(54.00)		54.69%	58.02%	
UUS	35(1.41)	14(3.39)		1.72%	1.86%	
AJCC Stage			0.000*			0.129
I	1632(65.70)	209(50.61)		63.35%	57.24%	
II	201(8.09)	73(17.68)		9.60%	10.42%	
III	205(8.25)	38(9.20)		8.36%	10.05%	
IV	446(17.95)	93(22.52)		18.69%	22.29%	
Lymphadenectomy			0.000*			0.767
Yes	819(32.97)	181(43.83)		34.61%	35.42%	
None/unknown	1665(67.03)	232(56.17)		65.39%	64.58%	
Peritoneal Cytology			0.004*			0.913
Negative	911(36.67)	184(44.55)		37.74%	38.05%	
Positive	95(3.82)	19(4.60)		3.94%	3.55%	
Unknown	1478(59.50)	210(50.85)		58.32%	58.40%	
Tumor size (mm)			0.069			0.378
50–100	862(34.70)	156(37.77)		35.21%	35.85%	
<50	420(16.91)	55(13.32)		16.34%	13.45%	
>100	892(35.91)	162(39.23)		36.42%	39.63%	
Unknown	310(12.48)	40(9.69)		12.03%	11.06%	
Chemotherapy			0.000*			0.170
Yes	937(37.72)	229(55.45)		40.46%	44.28%	
No	1547(62.28)	184(44.55)		59.54%	55.72%	
Radiotherapy						
Yes						
No						
Median income			0.178			0.910
$50,000–$65,000	847(34.10)	151(36.56)		34.47%	34.97%	
<$50,000	283(11.39)	56(13.56)		11.72%	12.21%	
>$60,000	1354(54.51)	206(49.88)		53.81%	52.82%	
Rural-urban			0.393			0.643
Rural	209(8.41)	40(9.69)		8.64%	9.48%	
Urabn	2275(91.59)	373(90.31)		91.36%	90.52%	
Months from DX to treatment			0.003*			0.979
<1	1886(75.93)	285(69.01)		74.93%	75.00%	
≥1	598(24.07)	128(30.99)		25.07%	25.00%	

Others *American Indian/Alaskan Native, Asian/Pacific Islander; *P* value with two asterisks indicates significantly statistical difference. DX, diagnosis; ESS, endometrial stromal sarcoma; LMS, leiomyosarcoma; UUS, undifferentiated uterine sarcoma.

### Univariate and multivariate analysis for cause-specific survival and overall survival

Prior to PSM and IPTW-adjustment, receipt of RT was associated with detrimental CSS (HR 1.17, 95% CI 1.00–1.36) and OS (HR 1.15, 95% CI 0.99–1.33) effect on univariate analysis (UVA), however, improved CSS (HR 0.80, 95% CI 0.68–0.94) and OS (HR 0.79, 95% CI 0.67–0.92) outcome on multivariate analysis (MVA), both with statistical significance. Based on UVA and MVA, chemotherapy showed detrimental effect on CSS and OS (HR > 1, *P* < 0.001). Factors associated with worse CSS and OS were patients older than 60 years, black race, single or unmarried status, higher tumor stage and grade, positive peritoneal cytology, tumor size bigger than 50 mm. Worse CSS and OS were also seen in patients administered with chemotherapy. Similar results were obtained following PSM and IPTW-adjustment by RT. CSS and OS improvements in patients who underwent RT persisted, as did the CSS and OS detriments associated with all other significant factors pre-adjustment. Adjusted and unadjusted UVA and MVA were shown in Table 3 and Supplementary Table S1, respectively.

**Table 3:** Univariate and multivariate analysis of predicting CSS and OS after IPTW-adjusted in stage I-IV US patients.

Characteristics	Cause-specific survival				Overall survival			
	Univariate analysis		Multivariate analysis		Univariate analysis		Multivariate analysis	
	HR (95% CI) *P*	*P*	HR (95% CI) *P*	*P*	HR (95% CI) *P*	*P*	HR (95% CI) *P*	*P*
Age (years)								
50–60	Reference		Reference		Reference		Reference	
<50	0.54(0.47–0.63)	<0.001*	0.70(0.60–0.82)	<0.001*	0.55(0.47–0.63)	<0.001*	0.70(0.60–0.81)	<0.001*
>60	1.12(0.98–1.28)	0.091	1.08(0.94–1.24)	0.302	1.21(1.07–1.38)	0.003	1.15(1.00–1.31)	0.049
Year of diagnosis								
2010–2012	Reference				Reference			
2013–2015	0.94 (0.82–1.08)	0.398			0.97 (0.85–1.11)	0.622		
2016–2018	0.97 (0.83–1.13)	0.672			0.99 (0.85–1.15)	0.871		
Marital status								
Divorced/separated	Reference		Reference		Reference		Reference	
Married	1.04(0.85–1.27)	0.689	0.99(0.82–1.22)	0.992	1.02(0.84–1.23)	0.858	0.98(0.80–1.19)	0.806
Single/unmarried	1.33(1.07–1.64)	0.011	1.27(1.02–1.58)	0.031	1.27(1.04–1.57)	0.022	1.23(1.00–1.52)	0.049
Unknown	1.10(0.81–1.51)	0.538	1.15(0.84–1.58)	0.397	1.07(0.79–1.45)	0.647	1.12(0.83–1.53)	0.454
Widowed	1.41(1.07–1.86)	0.015	1.23(0.93–1.63)	0.153	1.51(1.17–1.96)	0.002	1.29(0.99–1.68)	0.063
Race								
Black	Reference		Reference		Reference		Reference	
White	0.72(0.62–0.83)	<0.001*	0.80(0.69–0.93)	0.004	0.71(0.62–0.82)	<0.001*	0.80(0.70–0.92)	0.002
Others	0.58(0.46–0.73)	<0.001*	0.71(0.56–0.90)	0.005	0.60(0.48–0.74)	<0.001*	0.73(0.58–0.91)	0.006
Tumor grade								
I	Reference		Reference		Reference		Reference	
II	2.451(1.58–3.79)	<0.001*	2.28(1.46–3.56)	<0.001*	2.05(1.38–3.05)	<0.001*	1.90(1.26–2.87))	0.002
III	12.20(8.67–11.17)	<0.001*	7.60(5.23–11.03)	<0.001*	10.21(7.53–13.83)	<0.001*	6.52(4.66–9.11)	<0.001*
IV	11.77(8.48–16.35)	<0.001*	6.96(4.88–9.93)	<0.001*	9.54(7.14–12.75)	<0.001*	5.76(4.9–7.91)	<0.001*
Unknown	7.02(5.06–9.75)	<0.001*	5.02(3.50–7.19)	<0.001*	5.76(4.316–7.69)	<0.001*	4.17(3.02–5.75)	<0.001*
Histology								
Adenosarcoma	Reference		Reference		Reference		Reference	
ESS	1.21(0.94–1.55)	0.134	1.31(1.01–1.72))	0.042	1.11(0.88–1.40)	0.36	1.22(0.96–1.57)	0.106
LMS	2.68(2.15–3.34)	<0.001*	1.24(0.97–1.57)	0.085	2.41(1.96–2.95)	<0.001*	1.16(0.93–1.45)	0.186
UUS	3.19(2.07–4.92)	<0.001*	1.21(0.77–1.90)	0.403	3.00(2.00–4.51)	<0.001*	1.18(0.77–1.80)	0.442
AJCC Stage								
I	Reference		Reference		Reference		Reference	
II	1.85 (1.51–2.27)	<0.001*	1.65(1.33–2.04)	<0.001*	1.82 (1.50–2.22)	<0.001*	1.65(1.34–2.02)	<0.001*
III	3.67(3.04–4.39)	<0.001*	2.38(1.95–2.89)	<0.001*	3.44(2.88–4.12)	<0.001*	2.30(1.90–2.78)	<0.001*
IV	4.94 (4.31–5.66)	<0.001*	2.99(2.56–3.50)	<0.001*	4.72 (4.14–5.38)	<0.001*	2.98(2.56–3.47)	<0.001*
Lymphadenectomy								
None/unknown	Reference				Reference			
Yes	0.93 (0.82–1.05)	0.264			0.93 (0.83–1.05)	0.221		
Peritoneal Cytology								
Negative	Reference		Reference		Reference		Reference	
Unknown	1.10 (0.97–1.25)	0.132	1.01(0.89–1.14)	0.921	1.07 (0.95–1.21)	0.239	0.9 (0.88–1.12)	0.926
Positive	2.99 (2.34–3.82)	<0.001*	1.62(1.26–2.09)	<0.001*	2.99 (2.37–3.79)	<0.001*	1.64(1.29–2.09)	<0.001*
Tumor size (mm)								
50–100	Reference		Reference		Reference		Reference	
<50	0.43 (0.33–0.54)	<0.001*	0.68(0.53–0.87)	0.002	0.48 (0.39–0.60)	<0.001*	0.75(0.60–0.94)	0.013
>100	1.96 (1.72–2.23)	<0.001*	1.32(1.15–1.52)	<0.001*	1.92 (1.69–2.18)	<0.001*	1.31(1.15–1.50)	<0.001*
Unknown	0.78 (0.63–0.97)	0.022	0.92(0.73–1.14)	0.436	0.76 (0.62–0.94)	0.011	0.90(0.73–1.11)	0.326
Chemotherapy								
No	Reference		Reference		Reference		Reference	
Yes	3.31 (2.93–3.73)	<0.001*	1.30(1.13–1.50)	<0.001*	3.11 (2.77–3.49)	<0.001*	1.27(1.11–1.46)	0.001
Radiotherapy								
No	Reference		Reference		Reference		Reference	
Yes	1.14 (0.98–1.34)	0.093	0.83(0.96–1.25)	0.029	1.13 (0.97–1.33)	0.126	0.81(069–0.95)	0.011
Median income								
$50,000–$65,000	Reference				Reference			
<$50,000	0.98(0.81–1.19)	0.815			1.01(0.83–1.21)	0.958		
>$65,000	0.94(0.83–1.07)	0.340			0.95(0.84–1.08)	0.443		
Rural-urban area								
Rural	Reference				Reference			
Urabn	1.13(0.91–1.40)	0.266			1.12(0.91–1.37)	0.294		
Months from DX to treatment								
<1	Reference		Reference		Reference		Reference	
≥1	1.33(1.17–1.51)	<0.001*	1.09(0.96–1.25)	0.195	1.32(1.17–1.50)	<0.001*	1.07(0.94–1.22)	0.299

Inverse probability of treatment weighting (IPTW)-adjusted univariate and multivariable analysis. UVA included all variables and MVA included those with *P* < 0.1 on UVA.

* A hazard ratio (HR) of <1 favors surgery followed by RT and HR > 1 favors hysterectomy without RT given.

### Exploratory subgroup analysis in stage I-IV patients

**Figure 2:**
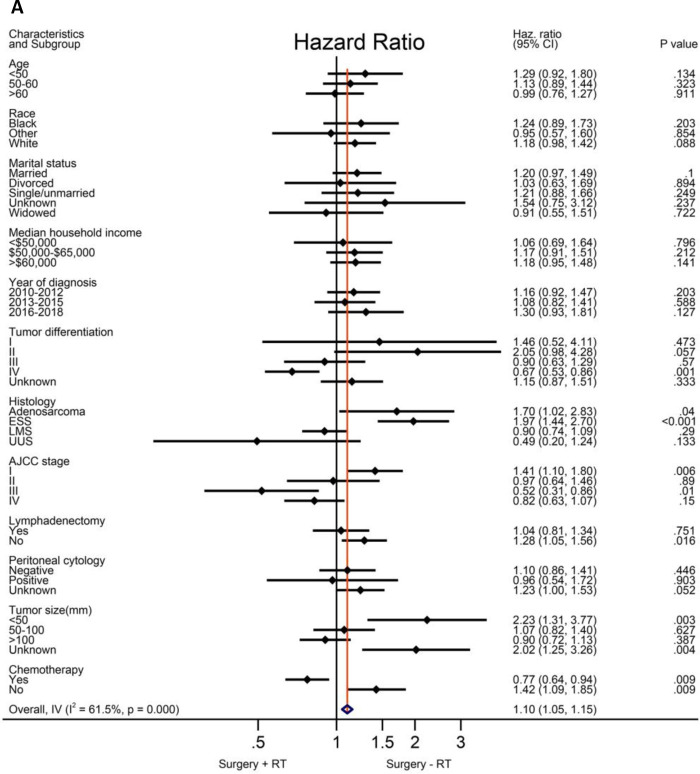
Exploratory subgroup analysis concerning radiotherapy impact on survival outcome in the whole cohort. (**A**) Cause-specific survival before IPTW-adjustment. (**B**) Overall survival before IPTW-adjustment. (**C**) Cause-specific survival after IPTW-adjustment. (**D**) Overall survival after IPTW-adjustment. CI: confidence interval; HR: hazard ratio; LMS: leiomyosarcoma; ESS: endometrial stromal sarcoma; USS: undifferentiated uterine sarcoma; IPTW: inverse probability of treatment weighting. The vertical solid-line refers to a hazard ratio of 1.0. HR < 1 favors surgery followed by radiotherapy and HR > 1 favors surgery without radiotherapy administered. *P* < 0.05 indicates statistical significance.

Based on the above analysis, radiotherapy showed beneficial effect of survival outcome, which promoted us to further explore who will finally benefit from radiotherapy administration. An exploratory subgroup analysis was conducted as shown in the forest plot (Figure 2). Before matching, heterogeneity was high (I^2^ > 50%) on fixed-effects model, interestingly, after matching by RT, heterogeneity was evidently decreased in both CSS and OS analysis (I^2^ < 50%). Therefore, we explored the fixed-effects model to illustrate the result. Prior to IPTW- adjustment, there were several subgroups with possible improved CSS (Figure 2A) and OS (Figure 2B) after RT administration, including patients older than 60 years of age, tumor grade III-IV, AJCC stage II-IV, LMS and UUS histology, positive peritoneal cytology, tumor size bigger than 100 mm, and those given with adjuvant chemotherapy. After IPTW-adjustment, improved CSS (Figure 2C) and OC (Figure 2D) were persistently observed in patients with LMS and UUS, tumor grade IV, AJCC stage III-IV, tumor size bigger than 100 mm, and with chemotherapy administration, although only patients in stage III showed statistical significance (*P* < 0.05).

**Figure:**
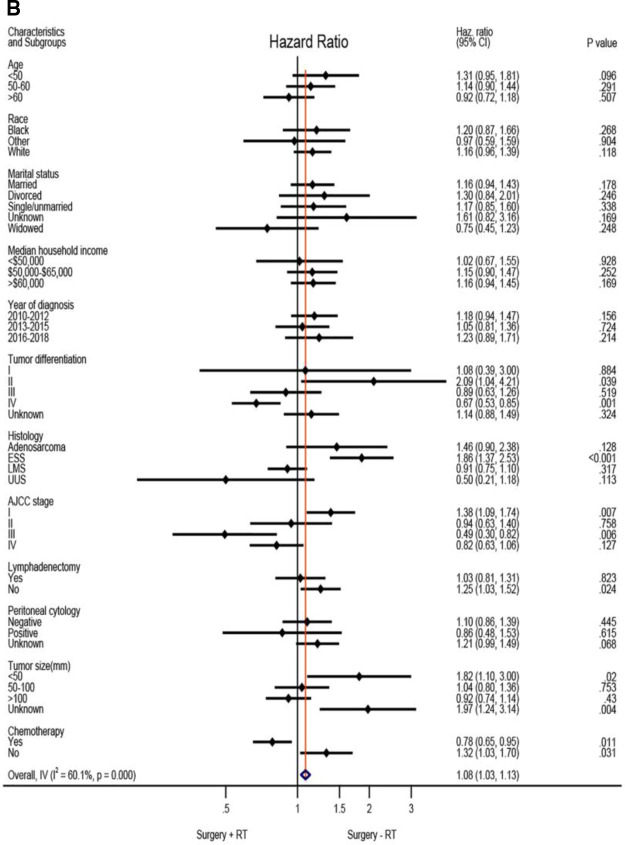


**Figure:**
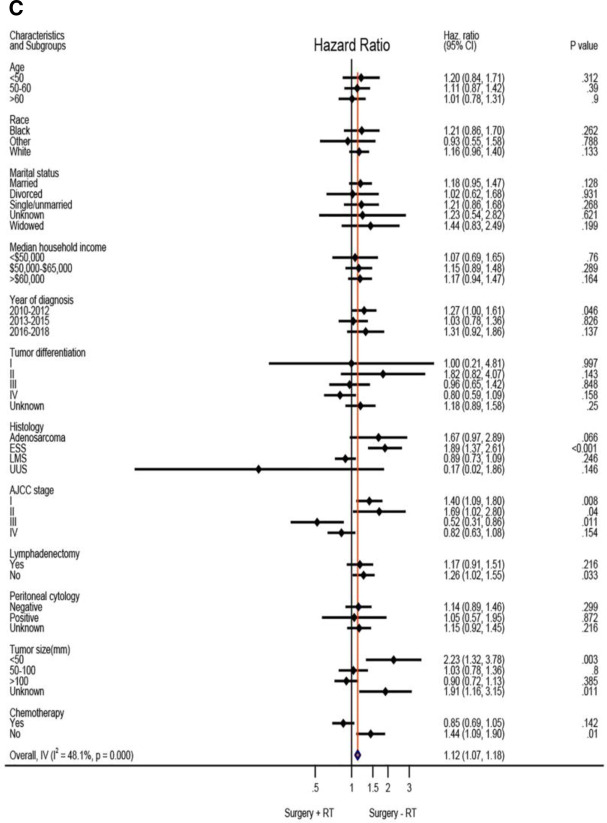


**Figure:**
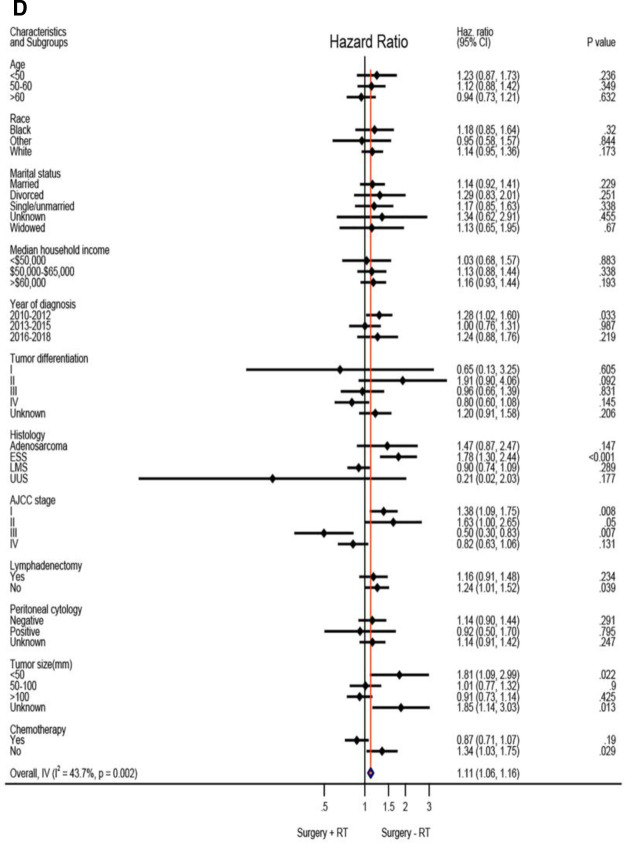


### Cause-specific survival analysis for stage II-IV US patients in selected subgroups

**Figure 3:**
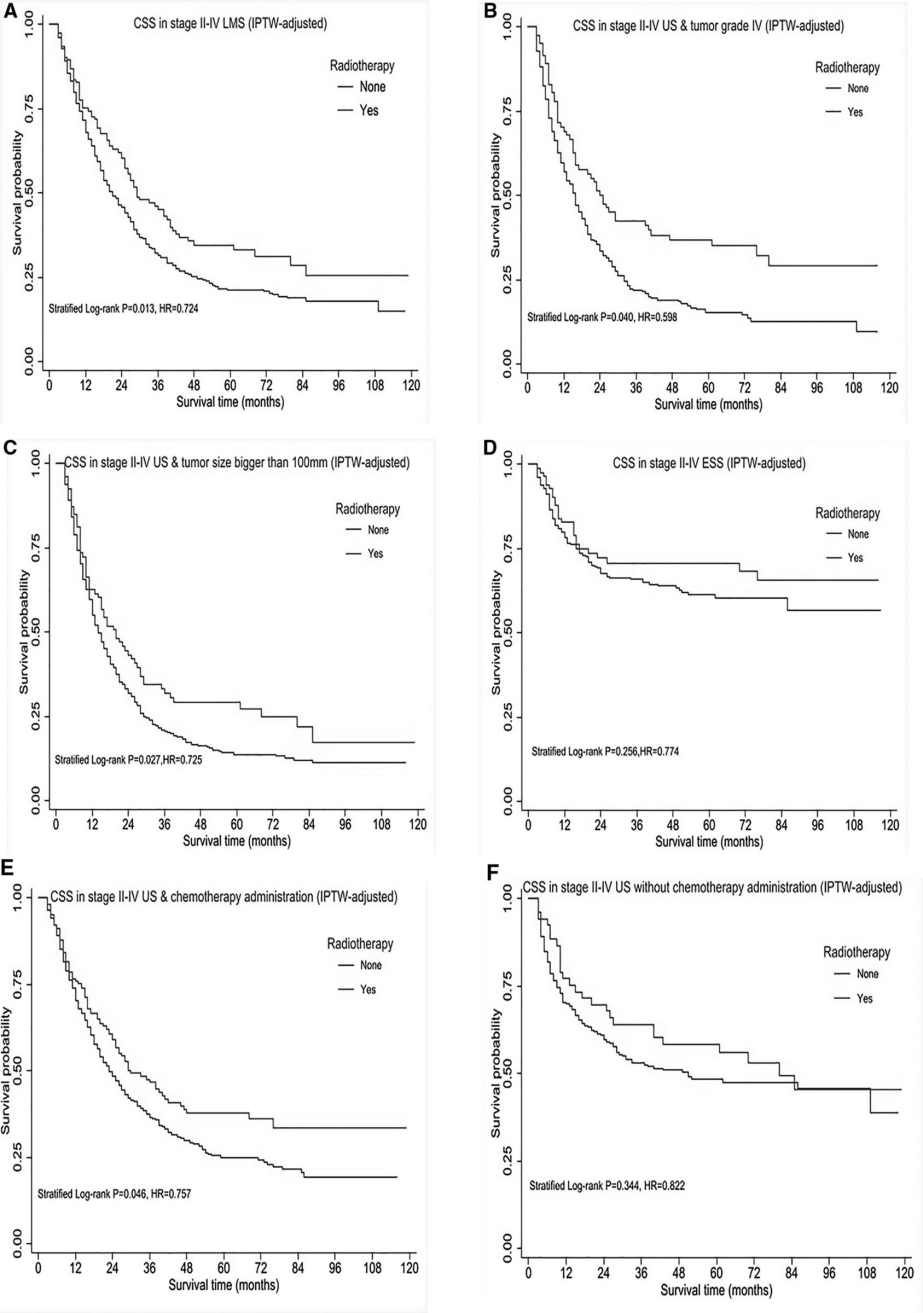
Subgroup survival analysis of cause-specific survival (CSS) in stage II-IV uterine sarcomas, after IPTW-adjustment by receipt of postoperative radiotherapy. (**A**) CSS in stage II-IV uterine leiomyosarcoma. (**B**) CSS in stage II-IV uterine sarcomas with tumor grade IV. (**C**) CSS in stage II-IV uterine sarcomas with tumor size bigger than 100mm. (**D**) CSS in stage II- IV ESS across all tumor grades. (**E**) CSS in stage II- IV uterine sarcomas treated by chemotherapy with/without radiotherapy. (**F**) CSS in stage II-IV uterine sarcomas without chemotherapy administration. HR < 1 favors surgery followed by radiotherapy and HR > 1 favors surgery without radiotherapy administered. *P* < 0.05 indicates statistical significance.

As demonstrated above, patients in stage II-IV possibly benefit from RT administration, interestingly, similar effect in CSS and OS. Therefore, we further explored RT impact on CSS in specific subgroups. A total of 1056 patients were identified within the stage II-IV cohort, of whom, 664 patients were diagnosed with LMS, 346 cases presented with tumor grade IV, 570 cases had bigger tumor size (>100 mm) and 694 patients received chemotherapy as part of their partial treatment. After IPTW-adjustment, patients in stage II-IV, particularly with LMS histology, tumor grade IV, tumor size bigger than 100 mm had improved CSS (Figures 3A–C, HR < 1, *P* < 0.05), however, no improvement in ESS patients (Figure 3D) across all tumor grades. Moreover, we performed survival analysis for USS in combination with HG-ESS (grade III/IV) at stage II-IV given the limited number of USS, and observed RT use could improve survival outcome. Among those cases who received chemotherapy, there was also improvement in CSS (Figure 3E). In contrast, no survival improvement was observed after RT given alone without CHT (Figure 3F).

## Discussion

Using the population-based, cancer registry SEER database and restricting the analysis to more recent period between 2010 and 2018, we gradually demonstrated a substantial survival improvement for high-risk patients with uterine sarcomas, when incorporating radiotherapy as an integral part following total hysterectomy. Most importantly, this benefit remained significant after stabilized IPTW adjustment to control for confounding factors and conditional landmark analysis, reducing the possibility that this conclusion suffered from selection bias and immortality bias, respectively. To our known, our analysis was the most up-to date, the first attempt to account for comprehensive confounding factors, and also encompassed a relatively wide spectrum of histological subtypes of uterine sarcomas.

Although chemotherapy was given in nearly 40% of patients in the present study, detrimental effect was observed at each specific follow-up period. The result promoted us to account for chemotherapy as a confounding factor and then focused radiotherapy impact on survival improvement, thus the corresponding results representing better balance between patients with or without adjuvant radiotherapy administration. Furthermore, exploratory subgroup analysis suggested the benefit of radiotherapy trended towards significance among the subgroup of patients receiving chemotherapy, although chemotherapy was found to be related to detrimental effect compared to no chemotherapy administration. This controversy might be explained by that chemotherapy was usually given in high-risk patients with poor prognosis. The subgroup analysis also indicated adjuvant radiotherapy was of detrimental for women with stage I disease in comparison to those who were treated with surgery alone, consistent with one previous largest SEER report regarding RT impact on uterine sarcomas patients (13). It is worth mentioning that RT was suggestive of potential benefit for patients with poor prognostic factors such as stage II-IV, grade IV, bigger tumor, in particular, patients with uLMS may benefit most from radiotherapy.

In cases of uterine LMS, due to its high recurrence rates (45%–75%) and extremely low 5-year survival rate (10%–15%) in metastatic disease (22), there has been great interest in exploring adjuvant therapy following surgery to reduce the risk of recurrence and improve survival. Yet, the utility of adjuvant RT has long been debated, given the majority of literature addressing the problem limited to retrospective reviews. The highest level of evidence from one prospective trial, the European Organization for Research and Treatment of Cancer (EORTC) protocol 55874, evaluated the impact of adjuvant RT on patients with stage I or II uterine sarcomas. As expected, the subgroup analysis of patients with uterine LMS indicated no benefit from RT in achieving either overall survival or local control, conversely a trend towards shorter OS period in the RT arm (23). Mahdavi et al. (24) investigated 147 patients with uterine LMS reported from 11 regional medical centers from 1985 to 2005 and then found the 5-year survival of patients who undertook radiotherapy was significantly higher than those who did not (70% vs. 35%); however, the survival advantage was no longer evident at 7.5 years. In addition, the local recurrence rate was lower in the radiotherapy group. The French Sarcoma Group compared adjuvant chemotherapy followed by RT with RT alone in surgically removed stage I-III uterine sarcomas including LMS. The 3-year DFS was 55% for adjuvant chemoradiotherapy vs. 41% for RT alone. Unfortunately, the study was prematurely closed for accrual futility (25). The above three studies either did not balance baseline characteristics to account for the receipt of chemotherapy, or not perform subgroup analysis in a wide perspective. Determining optimal adjuvant therapy is further confused in that stage II patients are often grouped with stage I subjects in clinical trials. Considering a number of unmeasured confounders influenced RT use, our study adjusted the confounding factors, finally, identifying RT use could possibly improve OS and CSS in stage II-IV LMS patients. This conclusion was in accordance with both ESMO and NCCN guidelines, both of which concluded RT is not recommended for stage I uLMS and should be discussed with patients in cases with higher stages considering special risk factors, such as mitotic count, age and tumor necrosis (26, 27). Based on the limited literature, in the advanced stage that is incompletely removed or metastatic disease, radiotherapy is persistently valuable when used in palliative treatment to distant locations (15), although adjuvant systemic chemotherapy is usually administered for unresectable advanced or recurrent disease (28).

With regard to endometrial stromal sarcoma (ESS), which represents the most common stromal sarcoma after leiomyosarcoma by frequency, several changes have been made in its classification. According to the recent 2020 WHO classification (29), it is currently divided into four main categories: endometrial stromal nodules, low-grade ESS (LG-ESS), high-grade ESS (HG-ESS) and undifferentiated uterine sarcoma (USS). Recurrences develop in 23–59% of all patients with ESS, and 15%–25% of these patients die of recurrent disease (30). In particular, HG-ESS showed a poor prognosis, with the 5-year survival rate of approximately 25%–30%. More than 60% of UUS patients are diagnosed at advanced stage and associated with an extremely poor prognosis (11). Due to the rarity and diversity of histology, there is no consensus or high level of evidence to support RT use in ESS. Some retrospective studies reported external pelvic radiation in patients across various stages of disease with HG-ESS and UUS to decrease local recurrence and improve overall survival (30, 31), although did not affect OS and PFS for low grade histology (32). However, NCCN guideline recommended observation after total hysterectomy with bilateral salpingoopherectomy (TAHBSO) for patients with stage I ESS irrespective of tumor grade, for stage II-IV TAHBSO, anti-estrogen hormone therapy and external beam could be performed for LG-ESS, systematic therapy and/or external beam radiation therapy for HG-ESS. Given small sample of USS and similar poor prognosis between USS and HG-ESS, our study performed survival analysis for USS in combination with HG-ESS at stage II-IV, and observed RT use could improve survival outcome, although no statistical significance at stage II-IV ESS across all tumor grades. This discrepancy between our study and NCCN guideline could be explained by HG-ESS reintroduced in 2014 (33) and experienced several alterations before then, yet our study included patients from 2010. Another potential explanation is that UUS is an extremely rare uterine sarcoma and represents the exclusion of diagnosis, thus no clear distinction between HG-ESS and UUS, limiting our analysis for RT impact on UUS and HG-ESS separately.

Similar to other uterine sarcomas, the majority of uterine adenosarcoma present with stage I disease in our study. Previous studies often pooled adenosarcoma with malignant Müllerian mixed tumors or other uterine mesenchymal neoplasms in adjuvant treatment strategies. Consequently, adjuvant treatment regimens did not reach consensus in general, only few studies in the literature referring to adjuvant therapy after complete staging surgery. However, it has been stated that patients at low risk of disease recurrence required observation alone, whereas for high-risk patients chemotherapy may be recommended (1, 34). Notably, in the 2016 National Cancer Data Base study of Müllerian adenosarcomas, adjuvant radiotherapy were reported to associate with a decreased overall survival (35). Both UVA and MVA in our study suggested adenosarcoma of better CSS and OS compared to other histological subtypes, yet small number of stage II-IV adenosarcomas restricted our analysis of adjuvant therapy effect on survival outcome.

Moreover, we also identified other potential prognostic factors, for instance, positive peritoneal cytology and bigger tumor. Survival period of US patients with positive peritoneal cytology was significantly shorter compared with those with negative cytology results, which agreed with the recent SEER analysis that recommended routine cytology testing at surgical treatment (36). Interestingly, the subgroup analysis prior to matching found radiotherapy meaningful in those cases with malignant peritoneal cytology, although the significance was not evident after matching, likely due to underestimate of peritoneal cytology as a prognostic factor in uterine sarcoma. Concerning tumor size, it is well recognized that the cut-off value between stage IA and stage IB is defined as 5 cm, based on 2009 FIGO staging system for LMS and ESS (37). However, only 25% of LMS measure less than 5 cm, typically voluminous tumors with a mean diameter of 10 cm (5). Hence, we divided tumor size with the cut-off point of 50 and 100 mm, consequently, demonstrating tumor size bigger than 100 mm as a possible indicator for radiotherapy utilization.

Although we attempted to account for nonrandom selection of patients, we recognized several inherent methodological limitations in this retrospective database analysis. First, our data lacked detailed information regarding tumor margin status which could have influenced the decision and effect of adjuvant therapy. Second, due to the unavailability of information in the SEER database, specific details on RT dose and technique, the effect of course as well as regimen of chemotherapy, sequencing with respect to adjuvant, neoadjuvant, or coadministration with RT remain unknown. Additionally, the current SEER database did not provide accurate distinction between none versus unknown chemotherapy receipt. Third, the SEER database did not describe specific surgery procedure such as the person who performed the surgery as well as mode of surgery, open or minimal invasive. Fourth and most importantly, our analysis focused primarily on OS and CSS, without details concerning local recurrence and distant metastasis after initial treatment, which could have important implications for the impact of adjuvant therapy in this patient population.

## Conclusions

Uterine sarcomas raise many controversies in oncogynecological practice. The results of the present study, in a stepwise process, suggested adjuvant radiotherapy might be underutilized, and proper use of adjuvant radiotherapy combined with chemotherapy after surgery in advanced stage and high-risk patients might improve survival. Prospective trials exploring precision medicine based on molecular profiles are still needed to determine the optimal adjuvant therapy for this rare disease.

## Data Availability

The raw data supporting the conclusions of this article will be made available by the authors, without undue reservation.
