# Supplementary material for: The role of postoperative radiotherapy in patients with uterine sarcomas: A PSM-IPTW analysis based on SEER database
Source: Front Surg. 2022 Aug 9;9:985654. doi: 10.3389/fsurg.2022.985654 (PMC9395938; doi:10.3389/fsurg.2022.985654)
Supplement: Supplementary file 1 [file Table_1_v1.docx]

Supplementary Table 1 Univariate and multivariate analysis of predicting CSS and OS before IPTW-adjusted in stage I-IV US patients

|  | Cause-specific survival | |  | | Overall survival | |  | |  |
| --- | --- | --- | --- | --- | --- | --- | --- | --- | --- |
| Characteristics | Univariate analysis  HR (95% CI) P | | Multivariate analysis  HR (95% CI) P | | Univariate analysis  HR (95% CI) P | | Multivariate analysis  HR (95% CI) P | |  |
|  |  |  |  |  |  |  |  |  |  |
| Age (years) |  |  |  |  |  |  |  |  |  |
| 50-60 | Reference |  | Reference |  | Reference |  |  |  |  |
| <50 | 0.54(0.46-0.63) | <0.001** | 0.70(0.60-0.81) | <0.001** | 0.54(0.47-0.63) | <0.001** | 0.70(0.60-0.81） | <0.001** |  |
| >60 | 1.12 (0.98-1.29) | 0.089 | 1.08 (0.93-1.24) | 0.308 | 1.21 (1.07-1.38) | 0.003 | 1.15 (1.00-1.31) | 0.050 |  |
| Year of diagnosis |  |  |  |  |  |  |  |  |  |
| 2010-2012 | Reference |  |  |  | Reference |  |  |  |  |
| 2013-2015 | 0.95 (0.82-1.09) | 0.424 |  |  | 0.97 (0.85-1.11) | 0.653 |  |  |  |
| 2016-2018 | 0.97 (083-1.13) | 0.659 |  |  | 0.99(0.83-1.15) | 0.856 |  |  |  |
| Marital status |  |  |  |  |  |  |  |  |  |
| Divorced/separated | Reference |  | Reference |  | Reference |  | Reference |  |  |
| Married | 1.04(0.85-1.27) | 0.700 | 1.00(0.82-1.23) | 0.995 | 1.02(0.84-1.23) | 0.871 | 0.98(0.81-1.19) | 0.816 |  |
| Single/unmarried | 1.32(1.07-1.64) | 0.011 | 1.27(1.02-1.58) | 0.031 | 1.27(1.04-1.57) | 0.022 | 1.23(1.00-1.52) | 0.049 |  |
| Unknown | 1.09(0.79-1.49) | 0.591 | 1.15(0.83-1.57) | 0.403 | 1.06(0.78-1.44) | 0.703 | 1.12(0.83-1.52) | 0.461 |  |
| Widowed | 1.40(1.07-1.854) | 0.016 | 1.23(0.93-1.63) | 0.152 | 1.51(1.16-1.95) | 0.002 | 1.29(0.99-1.68) | 0.063 |  |
| Race |  |  |  |  |  |  |  |  |  |
| Black | Reference |  | Reference |  | Reference |  | Reference |  |  |
| White | 0.72(0.62-0.83) | <0.001** | 0.80(0.69-0.93) | 0.004 | 0.72(0.62-0.82) | <0.001** | 0.80(0.69-0.93) | 0.004 |  |
| Others | 0.59(0.47- 0.74) | <0.001** | 0.71(0.56-0.90) | 0.005 | 0.60（0.48-0.75) | <0.001** | 0.71(0.56-0.90) | 0.005 |  |
| Tumor grade |  |  |  |  |  |  |  |  |  |
| I | Reference |  | Reference |  | Reference |  |  |  |  |
| II | 2.45(1.59-3.79) | <0.001** | 2.28(1.46-3.57) | <0.001** | 2.05(1.38-3.16) | <0.001** | 1.91(1.27-2.87) | 0.002 |  |
| III | 12.15(8.63-17.09) | <0.001** | 7.59(5.23-11.02) | <0.001** | 10.17(7.51-13.78) | <0.001** | 6.52 (4.66-9.11) | <0.001** |  |
| IV | 11.74(8.46-16.31) | <0.001** | 6.97(4.89-9.94) | <0.001** | 9.52(7.12-12.72) | <0.001** | 5.77 (4.20-7.93) | <0.001** |  |
| Unknown | 7.05(5.08-9.79) | <0.001** | 5.02(3.50-7.19) | <0.001** | 5.78(4.32-7.72) | <0.001** | 4.17 (3.02-5.75) | <0.001** |  |
| Histology |  |  |  |  |  |  |  |  |  |
| Adenosarcoma | Reference |  | Reference |  | Reference |  | Reference |  |  |
| ESS | 1.21(0.95-1.55) | 0.128 | 1.31(1.01-1.71) | 0.042 | 1.12(0.89-1.41) | 0.348 | 1.23(0.96-1.57) | 0.105 |  |
| LMS | 2.71(2.17-3.38) | <0.001** | 1.24(0.97-1.57) | 0.083 | 2.43(1.98-2.98) | <0.001** | 1.16(0.93-1.45) | 0.183 |  |
| UUS | 3.20(2.08-4.93) | <0.001** | 1.22(0.78-1.91) | 0.378 | 3.09(2.00-4.52) | <0.001** | 1.19(0.78-1.82) | 0.418 |  |
| AJCC Stage |  |  |  |  |  |  |  |  |  |
| I | Reference |  | Reference |  | Reference |  |  |  |  |
| II | 1.86 (1.51-2.28) | <0.001** | 1.67 (1.35-2.07) | <0.001** | 1.83 (1.50-2.22) | <0.001** | 1.66 (1.36-2.04) | <0.001** |  |
| III | 3.68(3.07-4.42) | <0.001** | 2.38(1.96-2.90) | <0.001** | 3.47(290-4.14) | <0.001** | 2.30 (1.90-2.79) | <0.001** |  |
| IV | 4.97 (4.34-5.69) | <0.001** | 3.00 (2.57-3.51) | <0.001** | 4.75 (4.16-5.41) | <0.001** | 2.99 (2.57-3.48) | <0.001** |  |
| Lymphadenectomy |  |  |  |  |  |  |  |  |  |
| None/unknown | Reference |  |  |  | Reference |  |  |  |  |
| Yes | 0.93 (0.82-1.05) | 0.248 |  |  | 0.93 (0.82-1.04) | 0.207 |  |  |  |
| Peritoneal Cytology |  |  |  |  |  |  |  |  |  |
| Negative | Reference |  | Reference |  | Reference |  |  |  |  |
| Unknown | 1.10 (0.97-1.25) | 0.125 | 1.01 (0.89-1.15) | 0.888 | 1.08 (0.96-1.21) | 0.228 | 1.00 (0.88-1.13) | 0.952 |  |
| Positive | 2.97 (2.32-3.79) | <0.001** | 1.62 (1.26-2.08) | <0.001** | 2.98 (2.35-3.76) | <0.001** | 1.64 (1.69-2.09) | <0.001** |  |
| Tumor size (mm) |  |  |  |  |  |  |  |  |  |
| 50-100 | Reference |  | Reference |  | Reference |  |  |  |  |
| <50 | 0.42 (0.33-0.54) | <0.001** | 0.67 (0.53-0.86) | 0.002 | 0.48 (0.38-0.60) | <0.001** | 0.75 (0.60-0.94) | 0.013 |  |
| >100 | 1.97 (1.72-2.54) | <0.001** | 1.32 (1.15-1.52) | <0.001** | 1.92 (1.69-2.18) | <0.001** | 1.31 (1.15-1.50) | <0.001** |  |
| Unknown | 0.77 (0.62-0.96) | 0.020 | 0.41 (0.73-1.14) | 0.407 | 0.76 (0.62-0.94) | 0.010 | 0.89 (0.72-1.11) | 0.305 |  |
| Chemotherapy |  |  |  |  |  |  |  |  |  |
| No | Reference |  | Reference |  | Reference |  |  |  |  |
| Yes | 3.32 (2.94-3.741) | <0.001** | 1.30 (1.13-1.50) | <0.001** | 3.12 (2.78-3.50) | <0.001** | 1.27 (1.10-1.46) | 0.001 |  |
| Radiotherapy |  |  |  |  |  |  |  |  |  |
| No | Reference |  | Reference |  | Reference |  |  |  |  |
| Yes | 1.17 (1.00-1.36) | 0.054 | 0.80 (0.68-0.94) | 0.007 | 1.15 (0.99-1.33) | 0.078 | 0.79 (0.67-0.92) | 0.002 |  |
| Median income |  |  |  |  |  |  |  |  |  |
| $50,000 - $65,000 | Reference |  |  |  | Reference |  |  |  |  |
| <$50,000 | 0.98(0.81-1.20) | 0.871 |  |  | 1.01(0.84-1.22) | 0.902 |  |  |  |
| >$65,000 | 0.95(0.83-1.07) | 0.384 |  |  | 0.96(0.85-1.08) | 0.493 |  |  |  |
| Rural-urban area |  |  |  |  |  |  |  |  |  |
| Rural | Reference |  |  |  | Reference |  |  |  |  |
| Urabn | 1.12(0.90-1.39) | 0.305 |  |  | 1.11(0.90-1.36) | 0.334 |  |  |  |
| Months from DX to treatment |  |  |  |  |  |  |  |  |  |
| <1 | Reference |  | Reference |  | Reference |  | Reference |  |  |
| ≥1 | 1.32(1.16-1.51) | <0.001** | 1.09(0.96-1.25) | 0.194 | 1.32(1.17-1.50) | <0.001** | 1.07(0.94-1.22) | 0.298 |  |

Inverse probability of treatment weighting (IPTW)-adjusted univariate and multivariable analysis. UVA included all variables and MVA included those with P < 0.1 on UVA. ** A hazard ratio (HR) of < 1 favors surgery followed by RT and HR > 1 favors hysterectomy without RT given.
